# Update in ocular surface squamous neoplasia

**DOI:** 10.1590/1806-9282.2024S124

**Published:** 2024-06-07

**Authors:** Andreia Novelli, Ivana Lopes Romero-Kusabara, Maria Auxiliadora Monteiro Frazão

**Affiliations:** 1 Department of Oculoplastics – São Paulo (SP), Brazil.; 2 Department of Health Sciences – São Paulo (SP), Brazil.; 3 Department of Ophthalmology – São Paulo (SP), Brazil.

## INTRODUCTION

Ocular surface squamous neoplasia (OSSN) is an entity that comprises the spectrum of squamous neoplasia of the conjunctiva and cornea, which includes conjunctival intraepithelial neoplasia (CIN), corneal epithelial dysplasia, squamous cell carcinoma (SCC), and mucoepidermoid carcinoma^1^. It mimics common conjunctival and corneal surface pathologies, for instance, pinguecula, pterygium, conjunctival granulomas, and cysts. In addition, OSSN has a high potential to cause ocular damage and systemic morbidity^2^. For this reason, it is important to raise awareness among the population regarding adequate eye protection and early diagnosis by ophthalmologists of suspicious lesions.

## EPIDEMIOLOGY

The prevalence of ocular surface squamous neoplasia (OSSN) demonstrates global variations due to differences in risk factors^1^. In the United States, its incidence rate has been documented in the range of 0.03–1.9 cases per 100,000 individuals per year, predominantly affecting Caucasian men between the sixth and seventh decades of life^1^. On the other hand, on the African continent, a notably high incidence was observed in younger patients, varying between 3 and 3.4 cases per 100,000 individuals per year, with a distribution that does not demonstrate gender preference^3^.

## ETIOLOGY, RISK FACTORS, AND PATHOPHYSIOLOGY

The etiology of OSSN proves to be multifactorial in nature and encompasses a diversity of elements, with the patient's immunological status possibly being the most crucial factor^3^. The risk factors most associated with the emergence of OSSN include exposure to ultraviolet B (UVB) radiation, human papillomavirus (HPV) infection, immunosuppression, and xeroderma pigmentosum^1^. The increased incidence of OSSN in individuals living in geographic regions close to the Equator is widely documented, due to greater exposure to UVB radiation^1^. Additional evidence corroborating this association is the finding that the majority of lesions occur in the interpalpebral fissure, nasal, and temporal limbus, regions that are most exposed to sunlight^3^. Limbal epithelial crypts are concentrated in the nasal region and contain epithelial stem cell niches in the basal layer^4^. It is possible that these are the progenitor cells in the OSSN that, after being altered, spread toward the surface before later invading the basement membrane^4^. UVB radiation acts by directly damaging DNA through the production of pyrimidine dimers in addition to other specific mutations in the p53 tumor suppressor gene, allowing cells with damaged DNA to surpass the cell cycle control point^4^. HPV is recognized as causing intraepithelial damage that culminates in the development of squamous neoplasms, and its subtypes 16 and 18 are specifically associated with the genesis of neoplastic lesions on the ocular surface^5^. UV radiation has also been described as causing local and systemic photoimmunosuppression and being capable of reactivating latent viruses, such as HPV^4^. Patients with some degree of immunodeficiency, especially those infected with the human immunodeficiency virus (HIV), have a substantially increased risk, approximately 10 times greater, for the development of OSSN and often exhibit more unfavorable clinical outcomes after treatment^5^. An additional etiological factor relevant to the development of OSSN is the failure of the DNA repair mechanism, as observed in xeroderma pigmentosum^3^. Other risk elements include advanced age, male sex, hypopigmented features of hair and eyes, xerophthalmia, trauma to the ocular surface, smoking, and chronic exposure to petroleum products^1^. Vitamin A deficiency interferes with the integrity of the ocular surface, creating microabrasions through which HPV can invade the basement membrane and conjunctival epithelial cells, initiating a cycle of local cellular changes^4^.

## GENERAL PATHOLOGY AND HISTOPATHOLOGY

The current classification for OSSN encompasses all dysplastic and carcinomatous lesions that affect the ocular surface^3^. Benign OSSN comprises conditions such as pseudotheliomatous hyperplasia, benign hereditary intraepithelial dyskeratosis, and papillomas^2^. Preinvasive OSSN, also known as intraepithelial neoplasias (CIN), are subdivided into three categories: CIN I (mild dysplasia restricted to the lower third of the conjunctival epithelium), CIN II (moderate dysplasia extending to the middle third), and CIN III (severe dysplasia affecting up to the upper third of the conjunctival epithelium)^2^. Dysplasia that involves the entire thickening of the epithelium is called carcinoma in situ (CIS)^2^. Finally, invasive OSSNs have the ability to cross the epithelial basement membrane, invading the conjunctival stroma and adjacent structures, and include SCC and mucoepidermoid carcinoma, which are more aggressive and recurrent than SCC^3^.

## HISTORY

Many patients affected by OSSN may remain asymptomatic, and the diagnosis is often suspected during routine ophthalmological examinations^3^. However, some may report the presence of a raised mass in the conjunctiva, accompanied by symptoms such as ocular irritation, itching, and ocular hyperemia^3^.

## CLINICAL PRESENTATION

The typical clinical presentation of OSSN involves the identification of elevated and nodular lesions in the interpalpebral region^1^. These lesions may vary in color from grayish white to reddish, have irregular margins, and are often accompanied by visible blood vessels^2^. Benign OSSN generally presents a papillomatous appearance^3^. On the other hand, intraepithelial neoplasias (CIN) can take on a leukoplastic and/or gelatinous macroscopic appearance^3^. Lesions with leukoplastic characteristics demonstrate superficial hyperkeratinization, while gelatinous lesions tend to be reddish and well defined, and may be nodular or diffuse^3^. When OSSN affects the cornea, resulting from the spread of abnormal epithelial cells from the limbus, it appears as an avascular, translucent, opalescent lesion with a ground-glass appearance, usually with defined margins^3^. Conjunctival SCC shares similarities with CIN; however, the conjunctival lesion tends to be higher, has a plaque shape, and presents less mobility^3^. In such cases, the identification of feeding vessels suggests rupture and invasion of the epithelial basement membrane^3^. Finally, mucoepidermoid carcinoma, a more aggressive and recurrent variant, clinically mimics SCC and can develop anywhere on the ocular surface^3^.

## DIAGNOSIS

The diagnosis of OSSN is generally established based on a combination of clinical data, ophthalmological examination, macroscopic characteristics, complementary noninvasive tests, and histopathological analysis^6^. Diagnostic suspicion based on an ophthalmological examination with the identification of a lesion is the fundamental step towards an adequate investigation of the case^3^. Although it is considered an invasive procedure, the gold standard for diagnosing OSSN is histopathological analysis after performing a biopsy, which is the only approach that makes it possible to detect the level of tissue invasion of the lesion^7^. It is evident that interest in conservative diagnostic approaches has grown significantly, and noninvasive methods, such as the use of vital dyes, cytology, confocal microscopy, and anterior segment optical coherence tomography (OCT-SA), have been applied with remarkable precision in the characterization of these lesions^3^.

The application of vital dyes, such as rose bengal, methylene blue, and toluidine blue, provides support for the diagnosis of OSSN in a practical and efficient way^6^. Rose Bengal has the ability to highlight degenerated epithelial cells, while methylene blue is useful in identifying malignant lesions, although both lack specificity directed exclusively to OSSN^6^. Toluidine blue, an acidophilic dye, has the property of staining cells with a high mitotic rate and of accumulating between them, especially in tissues with limited cell adhesion. Although this test reveals high sensitivity (92% in diagnosing OSSN), its specificity is considerably lower (31%)^6,8,9^.

Cytology represents an additional diagnostic method for OSSN and can be exfoliative or by impression^6^. Impression cytology involves obtaining superficial cells using filter paper made of cellulose acetate, which is applied directly to the target lesion^6^. Although this technique offers notable benefits, such as its minimally invasive nature and a correlation of results that reaches up to 80% agreement with histopathological analysis samples, it has limitations, including the superficial capture of cells and the requirement for an experienced cytologist to analyze the results^6^.

Studies involving the application of in vivo confocal microscopy (IVCM) in the context of OSSN have demonstrated diverse results, with a notable overlap of features observed in benign and malignant lesions^6^. Although IVCM can occasionally play a useful and complementary role to histology, it should not be considered a reliable substitute for biopsy due to the inconsistency of its results^6^.

Anterior Segment Optical Coherence Tomography has emerged as an extremely relevant diagnostic tool, allowing the acquisition of high-resolution images of the superficial ocular layers in a noninvasive manner and without the need for direct contact with the globe^7^. Its distinctive characteristics in the assessment of OSSN include the identification of an abrupt transition between the healthy epithelium and the abnormal epithelium, in addition to revealing the anomalous thickening of this epithelium and the presence of hyper-reflectivity in the tumor region^7^. OCT-SA currently plays a prominent and unquestionably effective role in the precise differentiation between OSSN and benign conditions, as well as in the identification of other tumor entities^7^. This discrimination capacity has provided significant contributions to the diagnosis and clinical management of various ocular surface pathologies^7^.

## DIFFERENTIAL DIAGNOSIS

Due to the sharing of risk factors and because they are considered synchronous lesions, pterygia and pingueculae should be considered and remembered as important differential diagnoses of OSSN^10^. Other conditions that should be considered as potential differential diagnoses include amelanotic melanoma, corneal pannus, nodular corneal degeneration, pyogenic granuloma, sebaceous cell carcinoma, actinic keratosis, conjunctival cysts, and Bitot's spots, among other possible entities^6^. Due to overlapping clinical features, the differential diagnosis of OSSN can be challenging, and in many cases, additional evaluations may be necessary to confirm the diagnosis^6^.

## TREATMENT

Treatment aims to eliminate the lesion, prevent recurrences, and preserve vision when possible^3^. Therefore, early detection and smaller tumors will have a better prognosis. Although surgical excision is still the gold standard of treatment, conservative medical approaches have been more commonly used in recent years^3,11–13^.

Surgical excision of conjunctival lesions is performed following Shields “no touch” technique with the aim of avoiding the potential risk of seeding^14^. This technique involves wide tumor-free margins^3,15^, associated with cryotherapy on the remaining conjunctiva in a “double freeze, slow thaw.” The limbal application is avoided to prevent damage to limbal stem cell deficiency. The recommended duration of the contact is 3 s in one single application. Corneal lesions are removed through alcohol keratoepitheliectomy, while the scleral component is addressed with a partial lamellar sclerotomy. The residual conjunctival defect can be closed, primarily if the defect is less than 3 clock hours. In large defects (more than 3 clock hours), conjunctival autografts or amniotic membrane grafts may be used.

A biopsy, whether incisional (extensive lesions) or excisional, allows histopathological analysis and diagnosis. Enucleation or exenteration is reserved for cases with intraocular or periocular invasion, respectively.

The nonsurgical therapies include topical chemotherapy [mitomycin C (MMC) and 5-fluorouracil (5-FU)], injection/topical immunotherapy (interferon alpha-2b), topical antiviral medication (cidofovir), anti-vascular endothelial growth factor (anti-VEGF), or photodynamic therapy (PDT)^11,12^.

Topical therapy has some advantages that include treatment of the entire ocular surface (areas of subclinical disease) as well as a lower risk of conjunctival scarring. Besides, these therapies can be used as adjuvants both preoperatively (chemoreduction) and postoperatively (to complement the treatment when margins are positive for tumor).

Mitomycin C (MMC) is an alkylating agent with antineoplastic properties. It is toxic to proliferating and non-proliferating cells by inducing apoptosis and inhibiting the migration of fibroblasts. Both the regimens of 1 drop of MMC 0.02% three times daily for two 1-week courses^16^ and 1 drop of MMC 0.04% four times daily for two 1-week courses^17^ have been demonstrated to be effective. Some side effects include dry eye, punctal stenosis, persistent epithelial defects, and allergic reactions. Therefore, MMC is often reserved for more recalcitrant cases that have failed prior therapy with alternative agents.

It is known that 5-fluorouracil (5-FU) is a pyrimidine analog that blocks thymidine synthase, which inhibits DNA formation. It acts on the S phase of the cell cycle and has been delivered topically as a 1% 5-FU formulation four times daily for four weeks^18^ or for 1 week followed by a drug holiday of 3 weeks^19^. Primary therapy has shown an efficacy of 85–100%^18–20^ and a recurrence rate ranging from 1.1 to 43%^21^. The side effects of topical 5-FU are, mainly, pain and redness at the instillation side; however, these side effects are fewer than those of MMC. The 5-FU is used in conjunction with topical corticosteroids and preservative-free artificial tears to reduce the symptoms^12^.

Interferon-alpha 2b (IFN-α2b) is the immunotherapeutic agent used in the treatment of OSSN. Interferons correspond to natural glycoproteins with antimicrobial and antiviral properties^12^. Their role as an antineoplastic agent is due to their antiproliferative, antiangiogenic, and cytotoxic effects, as well as their property of being a potential inducer of the host antitumor immunosurveillance^22^. IFN-α2b can be used as the primary agent for small lesions, as a neoadjuvant agent for diffuse tumors with the aim of assisting in surgical resection, and as adjuvant therapy when the margins after resection were positive for the presence of tumors^11,23^. It may be prescribed in two ways: topically as drops or locally as perilesional subconjunctival injections. The dosage of topical IFN-α2b is 1 million IU/mL, one drop four times per day without interruption until one or two more months after clinical resolution of the lesion^12,24^. There is no consensus on the dosage of local IFN-α2b to be injected. Subconjunctival injections (3 million IU/0.5 mL) are administered weekly until OSSN resolution (generally, 4 or 5 injections are necessary for adequate treatment). The injections have more side effects than the drops (e.g., flu-like symptoms)^12,25^.

Cidofovir is an antiviral agent with activity against double-stranded DNA viruses, including HPV. The dose of 2.5 mg/mL topical cidofovir has shown good efficacy as a secondary treatment in multi-refractory OSSN^26^.

Anti-VEGF agents are monoclonal antibodies that block the interaction of VEGF and its receptor, interfering with the growth of blood vessels^27^. There have been a few case reports on the use of these agents as primary therapy in OSSN or as adjuvants after surgical excision; therefore, their role remains uncertain^28,29^.

## PROGNOSIS

The recurrence rate of OSSN after treatment is variable, and the excision margin at the time of surgery is cited as the most important factor in predicting recurrence^3^. In general, the prognosis of CIN with free surgical margins is favorable, associated with low rates of local recurrence^3^. However, when excision margins are inadequate, especially in large lesions (greater than 2 mm), in elderly patients, or when deep tissue or cornea involvement occurs, the tendency for recurrence increases, reaching up to one-third of cases^3^. It is important to note that invasive carcinoma and mucoepidermoid carcinoma, more aggressive variants of OSSN, have less favorable prognoses and higher rates of local recurrence, even when undergoing surgical treatment with free margins^3^. Furthermore, due to the possibility of late recurrences that can occur even after years of treatment, it is recommended that patients undergo regular and permanent monitoring, with annual consultations, in order to monitor possible recurrences of the disease^30^.
